# Plasticity of the Injured Human Spinal Cord: Insights Revealed by Spinal Cord Functional MRI

**DOI:** 10.1371/journal.pone.0045560

**Published:** 2012-09-19

**Authors:** David W. Cadotte, Rachael Bosma, David Mikulis, Natalia Nugaeva, Karen Smith, Ronald Pokrupa, Omar Islam, Patrick W. Stroman, Michael G. Fehlings

**Affiliations:** 1 Institute of Medical Science, University of Toronto, Toronto, Ontario, Canada; 2 Department of Surgery, Division of Neurosurgery, Toronto Western Hospital, Toronto, Ontario, Canada; 3 Krembil Neuroscience Centre, Toronto Western Hospital, University Health Network, Toronto, Ontario, Canada; 4 Centre for Neuroscience Studies, Queen’s University, Kingston, Ontario, Canada; 5 Department of Radiology, Division of Neuroradiology, Toronto Western Hospital, University of Toronto, Toronto, Ontario, Canada; 6 Providence Health Care, Spinal Rehabilitation Unit, Kingston, Ontario, Canada; 7 Division of Neurosurgery, Kingston General Hospital, Queen’s University, Kingston, Ontario, Canada; 8 Department of Radiology, Division of Neuroradiology and Head & Neck Imaging, Kingston General and Hotel Dieu Hospitals, Queen’s University, Kingston, Ontario, Canada; 9 Departments of Diagnostic Radiology and Physics, Queen’s University, Kingston, Ontario, Canada; 10 Division of Genetics and Development, Toronto Western Research Institute, Toronto Western Hospital, University Health Network, Toronto, Ontario, Canada; University of North Dakota, United States of America

## Abstract

**Introduction:**

While numerous studies have documented evidence for plasticity of the human brain there is little evidence that the human spinal cord can change after injury. Here, we employ a novel spinal fMRI design where we stimulate normal and abnormal sensory dermatomes in persons with traumatic spinal cord injury and perform a connectivity analysis to understand how spinal networks process information.

**Methods:**

Spinal fMRI data was collected at 3 Tesla at two institutions from 38 individuals using the standard SEEP functional MR imaging techniques. Thermal stimulation was applied to four dermatomes in an interleaved timing pattern during each fMRI acquisition. SCI patients were stimulated in dermatomes both above (normal sensation) and below the level of their injury. Sub-group analysis was performed on healthy controls (n = 20), complete SCI (n = 3), incomplete SCI (n = 9) and SCI patients who recovered full function (n = 6).

**Results:**

Patients with chronic incomplete SCI, when stimulated in a dermatome of normal sensation, showed an increased number of active voxels relative to controls (p = 0.025). There was an inverse relationship between the degree of sensory impairment and the number of active voxels in the region of the spinal cord corresponding to that dermatome of abnormal sensation (R^2^ = 0.93, p<0.001). Lastly, a connectivity analysis demonstrated a significantly increased number of intraspinal connections in incomplete SCI patients relative to controls suggesting altered processing of afferent sensory signals.

**Conclusions:**

In this work we demonstrate the use of spinal fMRI to investigate changes in spinal processing of somatosensory information in the human spinal cord. We provide evidence for plasticity of the human spinal cord after traumatic injury based on an increase in the average number of active voxels in dermatomes of normal sensation in chronic SCI patients and an increased number of intraspinal connections in incomplete SCI patients relative to healthy controls.

## Introduction

Traumatic spinal cord injury (SCI) results in an acute disruption of afferent somatosensory signals and an inability to process motor, autonomic and reflex arcs within the damaged region of the spinal cord. The effect of traumatic injury to the spinal cord is classically considered in two stages, the primary injury, whereby mechanical forces are transmitted through the spinal column resulting in a shear force to axons and blood vessels within the spinal cord and the secondary injury that encompasses a cascade of events whereby the remaining viable neural tissue responds to its new, damaged environment.[1] For example, traumatic disruption of the microvasculature and increased interstitial pressure can lead to hypoperfusion and subsequent ischemia of the spinal cord.[1], [2], [3] The secondary effects of this ischemic insult include cytotoxic cell swelling of both neurons and glial cells, which disrupts action potential transmission.[4] Despite secondary destructive mechanisms, the spinal cord has an innate ability to recover varying degrees of sensory, autonomic and motor function.[5] This interplay between ongoing destructive mechanisms and innate reparative processes eventually reaches a balance that is often described in terms of adaptive or maladaptive plasticity.

Whether a patient recovers along the spectrum of adaptive plasticity, or experiences the unfortunate consequences of maladaptive plasticity, is currently established only after clinical symptoms are apparent. Adaptive changes include recovery of sensation or motor or autonomic function, whereas maladaptive plasticity results in spasticity or neuropathic pain. A number of pre-clinical investigations are progressing toward a deeper understanding of the cellular mechanisms underlying plasticity[6], [7] and clinical trials are underway to mitigate the secondary mechanisms of injury.[8] In fact, a recent international clinical trial demonstrated that early decompressive surgery favors adaptive plasticity in the form of improved motor and sensory function at six months after injury.[9], [10] With non-invasive spinal fMRI methods, we hope to better characterize both the degree of neural activity in specific regions of interest (for example, the dorsal horn) and how spinal circuits function as a whole (connectivity) to process information in the setting of spinal cord injury. In this respect, it may be possible to monitor the effects of different treatment strategies that aim to promote adaptive rather than maladaptive plasticity. In addition, it may be possible to follow rehabilitation strategies and provide feedback with regard to the function of specific neuronal populations and how these spinal neurons function as a concerted network to transmit information to both the brain and the peripheral nervous system.

Evidence for plasticity of the central nervous system after traumatic SCI is abundant in animal models.[6], [11] Characterizing these changes in human patients is more challenging but has been accomplished with modern non-invasive imaging techniques. High-resolution structural magnetic resonance imaging (MRI), in the form of voxel-based morphometry measurements, has been used to demonstrate atrophy of the sensorimotor cortex following traumatic cervical SCI.[12] A brain fMRI study demonstrated a shift of cortical activation corresponding to tongue movements, toward the adjacent disconnected cortical hand area in chronic SCI patients, as compared to healthy controls.[13] Other studies concluded that somatotopic organization is left unchanged after SCI, but movements of muscle groups rostral to the site of injury result in increased activity in the primary motor cortex and associated regions of the cerebellum.[14] Using a longitudinal functional MRI design, we have also demonstrated temporal changes that occur in both the motor and sensory cortex that accompany recovery after SCI.[15].

Investigation of the spinal cord after traumatic injury is an inherently more difficult feat. Artifacts arising from magnetic susceptibility differences between bone and soft tissues, and cord motion, present challenges for conducting high quality studies in the spinal cord.[16] Nonetheless, neural activity has been detected using established spinal fMRI methods.[17], [18], [19] In addition, spinal fMRI has been used to study spinal cord injury where activity has been reported caudal to the site of injury.[20] This is an important contribution to the spinal literature as it demonstrates that neuronal populations below the level of injury are engaged. Spinal fMRI studies have also demonstrated a graded stimulus response pattern across cool – cold noxious stimuli below the level of SCI.[21].

Both animal and human spinal fMRI studies have shown that spinal processing of sensory input is altered after SCI but there remains a significant knowledge gap as to the nature of this plasticity in the human spinal cord.[21], [22] Given the spectrum of clinical symptoms after traumatic SCI, ranging from a complete lack of sensation to altered sensation to normal sensation, we hypothesize that the injured spinal cord processes somatosensory information differently than healthy controls. Unique to this work, we apply a stimulus both above and below the level of the spinal cord injury in dermatomes of normal and abnormal sensation. In doing so, and with the application of a connectivity analysis, we aim to better understand changes that occur to somatosensory processing pathways following spinal cord injury.

## Methods

All methodological protocols received approval from institutional ethics review boards at both the University of Toronto, University Health Network, and Queens University.

### Participants

All participants provided written, informed consent prior to the initiation of the study and were free to withdraw at any time. If participants were not able to provide written consent due to the nature of their spinal cord injury, verbal consent was given and recorded on the consent form in the presence of a caregiver. Healthy controls were recruited by means of posted information. SCI participants were recruited though the clinic of the senior investigator (MGF) at the Toronto Western Hospital of the University Health Network and collaborators (KS and RP) at Kingston General Hospital.

In this study we compared healthy control subjects (n = 20, 12 at Toronto and 8 at Kingston) to chronic (>1year after injury) spinal cord injury patients (n = 18, 13 at Toronto and 5 at Kingston). Control and SCI patient characteristics are listed in Table 1. We grouped chronic SCI patients according to degree of sensory loss according to the American Spinal Cord Injury Association (ASIA)[23] grade: A (complete injury, no preserved sensation below the level of injury); B,C,D (incomplete injury with some preserved sensation below the level of injury) and E (complete motor and sensory recovery after SCI).

**Table 1:** Demographic and clinical information of healthy control subjects and chronic spinal cord injury patients.

Healthy Control Subjects								
N = 20, Average age = 37 years, age range 23–62, M:F = 10:10								
Chronic SCI subjects								
Participant No.	Age	Sex	Level of Injury	Thermodeplacement	ASIA grade	Overall AIS SensoryScore (LT/PP)	AIS sensory score ofdermatome abovethe level of SCI	AIS sensory score ofdermatome belowthe level of SCI
1	39	M	C6	C5/C8	A	108(76/32)	8	0
2	31	F	C4	C3/C8	A	12(6/6)	8	0
3	36	M	C5	C4/C8	A	22(10/12)	8	0
4	27	F	C5	C5/C8	B	81(54/27)	8	3
5	24	F	C4	C4/C8	C	78(51/27)	8	4
6	60	M	C6	C5/C8	D	223(112/111)	8	8
7	29	M	C6	C5/C8	D	214(102/112)	8	6
8	19	M	C6	C5/C8	D	150(79/71)	8	6
9	74	M	C5	C3/C8	D	215(109/106)	8	7
10	41	F	C6	C5/C8	D	218(108/110)	8	5
11	56	M	C7	C4/C8	D	207(104/103)	8	8
12	50	M	C2	C4/C8	D	115(49/66)	8	0
13	63	M	C5	C3/C6	E	224	8	8
14	25	F	C7	C5/C8	E	224	8	8
15	55	M	C6	C5/C8	E	224	8	8
16	60	M	C6	C5/C8	E	224	8	8
17	21	F	C6	C5/C8	E	224	8	8
18	66	M	C6	C5/C8	E	224	8	8

abbreviations: ASIA: American Spinal Injury Association; Grade A: complete loss of motor and sensory function below the level of injury with no sacral preservation; Grade B: incomplete injury with preservation of sensory but not motor function below the level of injury; Grade C: incomplete injury with preservation of motor and sensory function below the level of injury and motor function is grade 3/5 or less in key muscles below the level of injury; Grade D: incomplete injury with preservation of motor and sensory function below the level of injury and motor function is grade 3/5 or more in at least half of key muscles below the level of injury; Grade E: recovery to normal motor and sensory scores; LT: light touch; PP: pin-prick.

**A**merican Spinal Injury Association **I**mpairment **S**cale (AIS) sensory scores were evaluated in all patients in order to compare the degree of spinal fMRI activation to a clinical measure of sensory preservation. Briefly, the AIS sensory score is a measure of light-touch (LT) and pin-prick (PP) sensation that can be applied to each sensory dermatome. For example, the C5 dermatome can be evaluated as normal (AIS = 8) through to a complete lack of sensory perception (AIS = 0) where each of LT and PP are rated as normal (2 points), abnormal (1 point) or absent (0 points) on both the right and left side of the body. The AIS sensory scale thus evaluates a total of 28 sensory dermatomes (7 cervical, C2–C8, 12 thoracic, 5 lumbar and 4 sacral) with a maximum score of 8 points in each dermatome for an overall maximum of 224 points. This easy-to-use sensory scoring system has become the standard clinical measure to evaluate spinal cord injury patients.[24].

### Statistical Power Calculation

We utilized the method of Murphy[25] to estimate the number of time points necessary in an individual fMRI dataset. Based on a temporal signal-to-noise ratio of >40 (measured), a desired significance of p = 0.001 (GLM analysis, see below) and an effect size of approximately ±2%, it is necessary to collect 48 time points. The GLM analysis yields the number of ‘active’ voxels whereby we account for multiple comparisons by only considering voxels that cluster in a group of 5 or more (10 mm^3^).

Individual person data was grouped as follows: healthy controls (n = 20), incomplete SCI (n = 9) and recovered SCI (n = 6). To compare regions-of-interest (in terms of the number of active voxels), we used a random-effects analysis of the 1^st^ level GLM analysis as previously reported.[26] Based on a two-sample Student’s t-test, a sample size of 6 in each group is sufficient to detect differences of one standard deviation at a significance level of p<0.05 and a sample size of 12 in each group is sufficient to detect differences at a significance level of p<0.01.

### Thermal Stimulation

Using an automated thermal delivery system, heat (44°C) was applied to both the right and left side of the body, as described previously.[27] Thermal stimulation was applied in an identical fashion to both healthy control and SCI participants although the dermatome stimulated depended on the individual subject (see table 1). A total of 4 heating thermodes were used on each participant. Dermatomes were heated in an interleaved fashion. In control subjects, the C5 and C8 dermatomes were heated on both the right and left side of the body. In SCI participants, we chose a dermatome above the level of SCI and a dermatome below the level of SCI. For three individuals, it was not possible to place one or both thermodes in the prescribed positions. For example, individual number 5 (see table 2) had sustained a C4 ASIA C spinal cord injury and had thermodes placed on the C4 and C8 dermatomes. According to our experimental design, it would have been preferred to place the above level thermode on the C2 or C3 dermatome. However, this individual could not tolerate the thermode being placed on her neck. In such special circumstances, we placed the thermode as close as possible to the desired region, in this case the C4 dermatome. We do not expect that these deviations from protocol would have an impact on statistical comparisons, as the instances in which we had to deviate from protocol (3 in total, individuals 4, 5 and 12) occurred in incomplete SCI patients whereby individuals had normal sensation in the region stimulated (see Table 1). As previously reported,[26] thermal stimulation across different sensory dermatomes of normal sensation yield consistent patterns of activity within the spinal cord. The stimulation paradigm was different for each of the four thermodes, and the four paradigms form a linearly independent set. The paradigms spanned 405 seconds, and consisted of 3 warm stimulation periods of 45 seconds duration, separated by rest periods (passive cooling to skin temperature) of 45 seconds or 67 seconds. The paradigms began and ended with rest periods with durations of 67 seconds or more.

**Table 2:** The spinal fMRI response of chronic SCI patients.

Region of Interest	Site of stimulation	Location of cluster	Coordinates of active cluster withhighest absolute T value (dorsal-ventral, left-right, rostral-caudal)	total number of active voxels:positive (signal change abovebaseline) negative (signalchange below baseline)	
**Individual No. 4 27 F, C5 ASIA B**					
C5	Right side	Right dorsal horn	4, −5, 66.5	Positive:	22
			6.6, −1.5, 70.2	Negative:	11
	Left side	Left dorsal horn	5, 2.5, 70.5	Positive:	10
			8, 0.5, 67	Negative:	3
C8	Right side	Right dorsal horn	N/A	Positive:	0
			N/A	Negative:	0
	Left side	Left dorsal horn	N/A	Positive:	0
			7, 2.5, 111	Negative:	4
**Individual No. 5: 24 F, C4 ASIA C**					
C4	Right side	Right dorsal horn	N/A	Positive:	0
			6.6, −3.5, 49.4	Negative:	22
	Left side	Left dorsal horn	N/A	Positive:	0
			5, 6.5, 51.5	Negative:	10
C8	Right side	Right dorsal horn	6.3, −4.3, 105.5	Positive:	23
			3.7, −2.5, 113.7	Negative:	11
	Left side	Left dorsal horn	N/A	Positive:	0
			5.4, 1.4, 109.2	Negative:	11
**Individual No. 6: 60** **M, C6 ASIA D**					
C5	Right side	Right dorsal horn	7, −3.5, 66	Positive:	12
			7, −1.4, 64.2	Negative:	6
	Left side	Left dorsal horn	8, 3, 69.5	Positive:	8
			6, 3.5, 72.5	Negative:	6
C8	Right side	Right dorsal horn	6, −3.3, 112.3	Positive:	3
			4.7, −2.5, 110.3	Negative:	4
	Left side	Left dorsal horn	3.7, 2.5, 117.3	Positive:	7
			N/A	Negative:	0
**Individual No. 7: 29** **M, C6 ASIA D**					
C5	Right side	Right dorsal horn	7.5, −2.3, 72.3	Positive:	12
			N/A	Negative:	0
	Left side	Left dorsal horn	4.6, −0.3, 71.9	Positive:	18
			5.7, 3.6, 75.9	Negative:	7
C8	Right side	Right dorsal horn	N/A	Positive:	0
			N/A	Negative:	0
	Left side	Left dorsal horn	7, 1.4, 107.8	Positive:	10
			3.5, −0.5, 105	Negative:	23
**Individual No. 8: 19 M, C6 ASIA D**					
C5	Right side	Right dorsal horn	7.8, −2, 69	Positive:	25
			6.5, −3, 68	Negative:	17
	Left side	Left dorsal horn	N/A	Positive:	0
			8, 0.5, 74	Negative:	22
C8	Right side	Right dorsal horn	6, −4.5, 115	Positive:	5
			N/A	Negative:	0
	Left side	Left dorsal horn	5.5, 3.5, 120	Positive:	20
			4, 5.5, 106	Negative:	7
**Individual No. 9: 74 M, C5 ASIA D**					
C3	Right side	Right dorsal horn	8, −2.5, 45	Positive:	8
			N/A	Negative:	0
	Left side	Left dorsal horn	N/A	Positive:	0
			4.3, 0.9, 45.9	Negative:	18
C8	Right side	Right dorsal horn	5, −2.5, 105.5	Positive:	8
			6.5, −2.5, 109	Negative:	8
	Left side	Left dorsal horn	N/A	Positive:	0
			N/A	Negative:	0
**Individual No. 10: 41 F C6 ASIA D**					
C5	Right side	Right dorsal horn	7, −1.5, 70	Positive:	6
			8, −1.5, 71	Negative:	10
	Left side	Left dorsal horn	6, 0.5, 70	Positive:	8
			6.7, 0.5, 65.7	Negative:	13
C8	Right side	Right dorsal horn	7, −1.6, 108.8	Positive:	5
			3.6, −4.6, 111.8	Negative:	3
	Left side	Left dorsal horn	6, 0.7, 107.7	Positive:	10
			7, 0.3, 109.3	Negative:	10
**Individual No. 11: 56 M, C7 ASIA D**					
C4	Right side	Right dorsal horn	N/A	Positive:	0
			N/A	Negative:	0
	Left side	Left dorsal horn	3.6, 5.1, 54.2	Positive:	10
			6, 6, 54	Negative:	13
C8	Right side	Right dorsal horn	7, −2.5, 108	Positive:	12
			N/A	Negative:	0
	Left side	Left dorsal horn	6, 3.5, 105.5	Positive:	4
			6, 5.5, 108.5	Negative:	4
**Individual No. 12: 50 M, C2 ASIA D**					
C4	Right side	Right dorsal horn	4.7, −4.9, 52.2	Positive:	32
			7.5, −1.5, 58	Negative:	8
	Left side	Left dorsal horn	4, 2.5, 59	Positive:	6
			5.3, 0.5, 59.3	Negative:	19
C8	Right side	Right dorsal horn	N/A	Positive:	0
			6.3, −3.5, 106.7	Negative:	18
	Left side	Left dorsal horn	4.7, 6, 113.7	Positive:	27
			4.6, 0.5, 113.6	Negative:	20

### fMRI Data Acquisition

Spinal fMRI data were collected on a 3T General Electric system (GE Healthcare, Waukesha, WI) at TWH and a 3T Siemens system (Siemens Healthcare, Erlangen, Germany) at Queen’s University using previously established methods based on the SEEP (Signal Enhancement by Extravascular Protons) contrast mechanism which takes advantage of changes in tissue water content concomitant to neural activity.[28], [29] A 3D volume that spanned from the T1 vertebra to above the thalamus was imaged repeatedly by means of a half-fourier single shot fast spin-echo (HASTE, Siemens; SSFSE, GE) imaging sequence. This sequence consisted of 79 echoes with a spacing of 5.4 msec, and the 7^th^ echo was acquired at the center of k-space to produce an echo time 38 msec determining the image contrast. Nine sagittal slices were acquired contiguously with repetition time of 9 seconds (1 sec/slice), a 28×21 cm field-of-view (FOV) with 1.5×1.5×2 mm resolution. A total of 48 functional volumes were acquired. The image quality was enhanced by means of spatial suppression pulses anterior to the spine, and motion compensating gradients in the head-foot direction.

### Data Analysis

Prior to analysis, lines were manually drawn to demark the anterior, posterior, right and left edges of the cord in each dataset. Furthermore, the pontomedullary junction (PMJ) and the C7/T1 disc was marked as reference points in the image. These reference lines were later used to aid the co-registration and normalization steps. Co-registration was applied to correct for bulk body movements, and a 3-pixel-wide boxcar function smoothing kernel was applied in the direction parallel to the long axis of the spinal cord.

**Figure 1:**
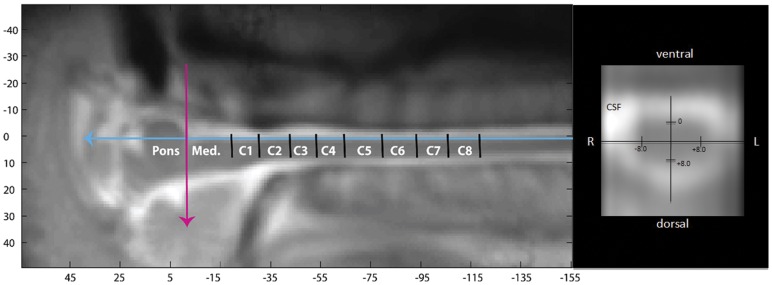
Spinal Coordinates. The above coordinate system was used to localize regions of the cervical spinal cord to the associated sensory dermatome. The pink arrow represents a rostral landmark – the ponto-medullary junction. The blue line represents the anterior border of the spinal cord. Shown on the right is an axial view with the white cerebrospinal fluid (CSF) along with the coordinates used to identify the ventral-dorsal and right-left divisions of the spinal cord. Zones of the spinal cord (C1 through C8) are labeled in white lettering and based on anatomic dissection of the cervical nerve rootlets as they emerge from the spinal cord (see text for further details and reference).

After aligning all fMRI data into a common coordinate system using the ponto-medullary junction as a rostral reference point, it was necessary to determine the location along the spinal cord where the cervical nerve roots enter/emerge from the cord corresponding to the dermatome stimulated. To do this, we used data obtained from a cadaveric dissection study that mapped out the rostral-caudal extent of cervical nerve rootlets and divided the spinal cord into zones.[30] After each zone was established in the common coordinate system, the spinal cord was further divided into four quadrants: right dorsal, right ventral, left dorsal and left ventral. This allowed for a region of interest analysis. For example, the dorsal right C5 region of the spinal cord corresponding to nerve rootlets serving the C5 dermatome can be found at the following coordinates: 8, −2.5, 45 whereby 8 is the distance in mm from the anterior edge of the spinal cord, −2.5 is the distance in mm from the midline (negative numbers represent right and positive number represent left) and 45 is the distance along the midline from the fixed reference point, the ponto-medullary junction (see figure 1).

Data were analyzed according to the general linear model (GLM).[31] The basis functions for the GLM consisted of the model paradigms which describe the presentation of the thermal stimulation, models of the cardiac-related motion [32], a linear ramp, and a constant function. The stimulation paradigms were then convolved with the hemodynamic response function, as determined in previous studies.[33] Normalization of the data was performed after the GLM was conducted on the data, for purposes of displaying group results. Significance was set at p<0.001. Correction for multiple comparisons was conducted by selecting only clusters of at least 5 active voxels (10 mm^3^). Within the GLM analysis, we captured both positive and negative correlations and termed these positive activation (a change in the fMRI signal above the resting baseline) and negative activation (change below the resting baseline) respectively. We did this so as to capture the entire change in spinal tissue consistent with thermal stimulation in the associated dermatome. While the precise biophysical events associated with negative activation remain somewhat elusive, a reduction in neuronal activity (and hence a reduction in the metabolic response) has been proposed.[28] It is important that the resting “baseline” condition involves tonic descending input to the spinal cord. Given the prominent role of inhibition in the spinal cord, the complexity of understanding and distinguishing positive and negative activation increases. While our study was not designed to test for differences between positive and negative activation signals, such work will be important for further characterizing the link between spinal fMRI signal change and the underlying physiology.

Connectivity analyses were applied to both individual and group average time-series data created from spatially normalized data from participants. A “seed” point was identified within each spinal cord region of interest by identifying the active voxel with the time-series with the highest correlation (positive activation), R, to the stimulation paradigm. For example, if the right C5 dermatome was stimulated, the seed point was chosen in the right dorsal quadrant of the C5 zone of the spinal cord. If no active voxels were detected in this region, then no connectivity was inferred. This seed point was grown into a “prime cluster” by identifying all of the contiguous voxels that are correlated with the seed point time-series at R ≥0.33. The average time-series of the cluster was then used for the connectivity analysis. Functional connectivity between the prime cluster and every other voxel in the brainstem and spinal cord was determined by means of the voxel-by-voxel correlation, with a threshold of R ≥0.5.

Group (healthy control, incomplete SCI, recovered SCI) differences of ROI activation, degree of connectivity (either inter-spinal or supra-spinal) and total number of connected voxels were analyzed by using a one-way ANOVA and post-hoc Tukey test (SPSS, Chicago IL). Differences in ROI activation were compared across a single spinal level. Correlation between clinical AIS sensory scores of the dermatomes stimulated (incomplete SCI) and the degree of ROI activation was carried out using Pearson’s correlation coefficient (SPSS, Chicago IL). A value of p<0.05 was assumed as the statistical threshold.

## Results

### Patient Characteristics & Anatomic Localization Along the Spinal Cord

In this study we compared 20 healthy individuals with 18 chronic traumatic spinal cord injury patients who sustained their injury at least 12 months prior to enrolling in our study. SCI patients were further categorized into sub-groups based on the severity of injury: complete (n = 3), incomplete (n = 9) and those that recovered full function (n = 6). Characteristics of this participant group are summarized in Table 1.

### Region of Interest Analysis: Healthy Controls

**Figure 2:**
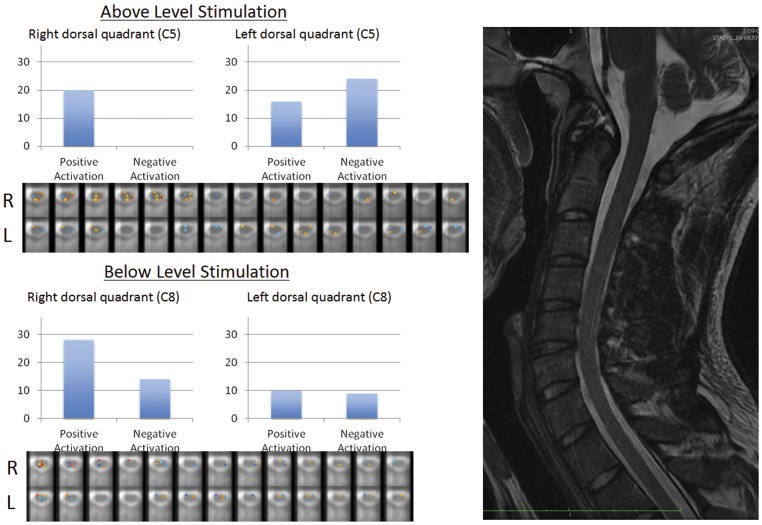
Single subject results. Shown is the spinal fMRI region of interest analysis of an uninjured 24-year-old male who underwent thermal stimulation of the C5 and C8 dermatomes on both the right and left side. The blue bar graphs represent the absolute number of active voxels contained within the dorsal quadrant each spinal cord zone. Positive indicates a change in proton density above the resting baseline and negative indicates a change below the resting baseline. Below each bar graph is the activation pattern across the stimulated region in axial cross-section. A T2 anatomical image is provided to the right for reference.

**Figure 3:**
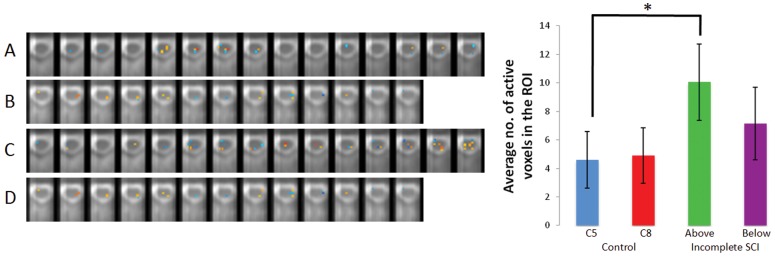
Region of interest analysis. Left panel: A region of interest (ROI) analysis conducted in a healthy individual (A and B) and a chronic SCI patient (C and D); axial images through the cervical zone of the spinal cord corresponding to the dermatome stimulated. A: Thermal stimulation of the right C5 dermatome of a 30 year old male. B: Thermal stimulation of the left C8 dermatome of the same individual. C: Thermal stimulation of the right C5 dermatome in a 19 year old male with a chronic C6 ASIA D SCI. D: Thermal stimulation of the left C8 dermatome in the same individual. Right: Quantification of the ROI analysis across all healthy controls (n = 20) and incomplete SCI patients (n = 9). Bar graph: uninjured controls stimulated at the C5 dermatome (blue) and C8 dermatome (red). Incomplete, chronic SCI patients stimulated above the level of injury (green) and below the level of injury (purple). A one way ANOVA was significant at p = 0.003. Post hoc tukey test revealed a significant difference between thermal stimulation above the level of injury in chronic SCI patients, *p = 0.025. There was no significant difference between thermal stimulation of the C5 and C8 regions of control participants. There was no significant difference between thermal stimulation below the level of injury in chronic SCI patients and thermal stimulation of either the C5 or C8 regions in healthy controls.

Thermal stimulation was applied to the C5 and C8 dermatomes on the right and left side of the body (a total of four stimulation points) for each of the healthy controls. The corresponding zone of the spinal cord was defined as the dorsal quadrant of that dermatomal segment (i.e. C5 right dermatome corresponds to the right C5 dorsal quadrant of the spinal cord). Spinal fMRI data sets were analyzed on a voxel-by-voxel basis using the general linear model according to the thermal stimulation paradigm for each dermatome. Those voxels showing a change in signal intensity above the baseline (positive activation) are displayed along the red spectrum (axial images, see figure 2) and those showing a change in signal intensity below the baseline (negative activation, see figure 2) are displayed along the blue spectrum. Figure 2 demonstrates an individual example whereby active voxels in the region of interest are shown along with the axial reconstructions of functional images. The average number of active voxels in the C5 region is 4.6±1.9 and the C8 region is 4.9±1.9 representing the entire group of healthy controls (n = 20). (See figure 3) There was no statistical difference between the average number of active voxels between upper and lower levels.

### Region of Interest Analysis: ASIA A Complete SCI

Our patient participants consisted of 3 individuals who sustained severe injuries and were left with no motor or sensory function below the level of their injury (ASIA A). When a dermatome of normal sensory perception was stimulated (above the level of injury), the average number of active voxels in the corresponding zone of the spinal cord was 14±9.4, not significantly different than the average number of active voxels in dermatomes of normal sensation in controls. There was a relatively high degree of variability in the ASIA A participant population, as evidenced by the 95% confidence interval. The zone of the cervical spinal cord corresponding to the dermatome stimulated below the level of injury was severely damaged in each of these 3 individuals (images not shown). Based on the extensive tissue damage in the area of interest, isolation of a functional signal change is of questionable significance and was not conducted.

### Region of Interest Analysis: Incomplete SCI (ASIA B, C, D) and Recovered SCI (ASIA E)

Our patient participants consisted of 9 individuals with an incomplete SCI (ASIA B, C, D) and 6 individuals who recovered completely from their SCI (ASIA E). Thermal stimulation was applied to a dermatome above and below the level of spinal cord injury. On an individual patient basis, the number of active voxels was greater in regions above the level of SCI in comparison to dermatomes below the level of SCI. (Individual patient data not shown; see table 2 for detailed information) On a group level, incomplete SCI patients (ASIA B, C, D), the average number of active voxels above the level of injury, in a dermatome of normal sensation, was 10.1±2.5 voxels and the average number of active voxels below the level of injury was 7.1±3.0 voxels (see figure 3). Table 2 summarizes the region of interest, site of thermal stimulation, coordinates of active voxels and the total number of active voxels for each individual. A one-way ANOVA comparing the mean number of active voxels in control participants and the mean number of active voxels in incomplete SCI patients was significant at p = 0.003. A post-hoc Tukey test revealed a significant group difference between control participants stimulated on the C5 dermatome and incomplete SCI participants stimulated above the level of their SCI in a dermatome of normal sensation (p = 0.025). There was no significant difference between incomplete SCI participants stimulated below the level of injury and that of healthy controls. The average number of active voxels in participants that recovered completely from their SCI (ASIA E) was 8.2±3.4 voxels above the level of the initial SCI and 10.5±3.8 voxels below the level of the initial SCI, not significantly different from either controls or incomplete SCI participants (data not shown).

**Figure 4:**
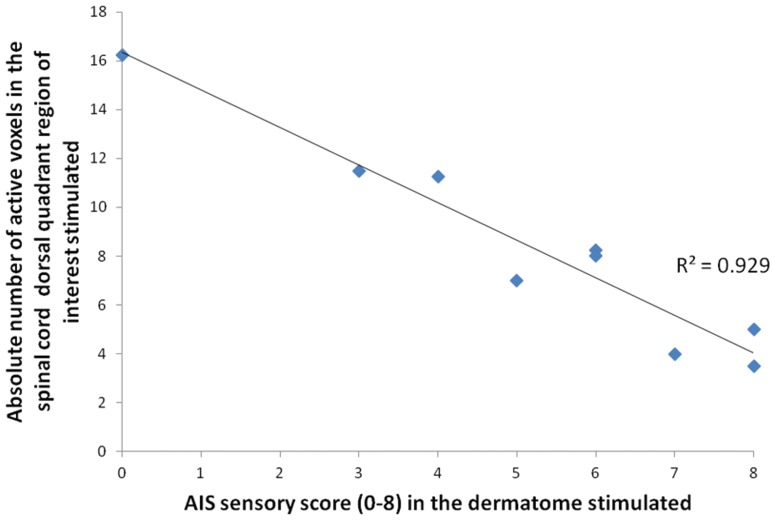
Spinal fMRI/sensory deficit relationship. Pearson’s correlation analysis between the absolute number of active voxels in the spinal cord dorsal quadrant region of interest (corresponding to the dermatome stimulated) and the AIS clinical sensory score of that same dermatome. All data contained within this analysis was taken from incomplete SCI patients who were stimulated in dermatomes below the level of their SCI. R^2^ = 0.93, p<0.001. AIS sensory score  = 8, normal sensation. AIS sensory score  = 0, no sensation. AIS sensory score encompasses both pin-prick and light touch clinical tests.

Next, we examined the relationship between the degree of spinal fMRI signal change and the degree of sensory impairment in participants with an incomplete SCI (ASIA B, C, D). To do this, we utilized Pearson’s correlation coefficient to compare the number of active voxels in the dorsal quadrant of the spinal cord that corresponded to the dermatome of abnormal sensation stimulated. For example, participant number 4 (ASIA B SCI) had a combined AIS sensory score of 3/8 in the C8 dermatome. There is a correlation between the AIS sensory score and the number of active voxels in the corresponding region of the spinal cord, R^2^ = 0.93, p<0.001 whereby more impaired regions of sensation (a low AIS sensory score) are associated with a higher degree of spinal fMRI activation and a normal region of sensation is associated with a lower degree of activation. The results are shown in Figure 4, dermatome sensory scores are reported in Table 1.

### Functional Connectivity Analysis

In order to determine the relationship between active voxels in the region of the spinal cord corresponding to the dermatome stimulated and the remainder of the spinal cord and brainstem, we applied a functional connectivity analysis. Functional connectivity was inferred from the temporal correlation between all voxels in a selected region of interest, and all other regions. For example, when the C5 dermatome is stimulated, we examined the relationship between the prime cluster in the C5 region of the spinal cord and other regions of the spinal cord and brainstem. In this analysis we examined three different variables: the number of interspinal connections (within the cervical spinal cord), the number of supra-spinal connections (connections between the region-of-interest and the brainstem) and the total number of connected voxels.

**Figure 5:**
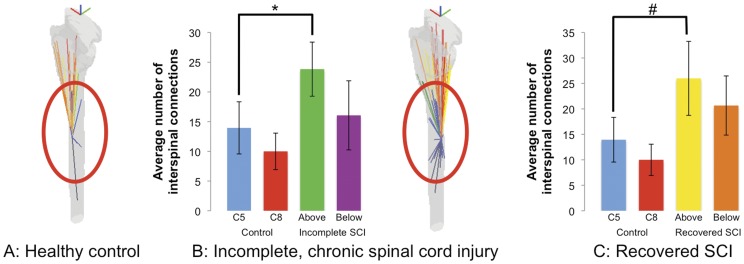
Spinal connectivity analysis. From left to right: Interspinal connectivity analysis in healthy controls (projected image), statistical comparison of controls to incomplete SCI patients (bar graph), interspinal connectivity analysis in incomplete SCI patient (projected image) and statistical comparison of controls to recovered SCI patients. The projected images represent a single subject from three angles (oblique left, coronal, obliqe right). The blue lines represent interspinal connections, the orange-yellow lines represent spinal cord-caudal brainstem connections and the red spectrum represents spinal cord-rostral brainstem connections. The number of inter-spinal connections to the prime cluster is shown as a projected image for both uninjured controls (A) and incomplete, chronic SCI patients (B). A one-way ANOVA comparing the mean number of interspinal connections to the prime cluster across healthy control (blue and red bar graph), incomplete SCI (green and purple bar graph) and recovered SCI participants (yellow and orange bar graph) was significant (p<0.001). Significant post-hoc Tukey tests included the difference between control participants stimulated in the C5 dermatome and incomplete SCI patients stimulated above the level of injury (* p = 0.045, blue vs. green bar graph) and recovered SCI participants stimulated above the level of injury (# p = 0.03, blue vs. yellow bar graph).

The number of interspinal connections was significantly higher in incomplete SCI patients stimulated above the level of their injury in a dermatome of normal sensation, p = 0.045, in comparison to healthy controls (figure 5, left bar graph). Similarly, in persons who recovered from their SCI (ASIA E), the number of interspinal connections is significantly higher in comparison to controls when stimulated above the level of injury (p = 0.03) (figure 5, right bar graph). The overall one-way ANOVA for the group comparisons was significant at p<0.001. There was no significant difference between control and SCI participants when stimulated below the level of injury. In addition, there was no significant difference between the controls and SCI participants in terms of supraspinal connections or the total number of connected voxels (data not shown).

## Discussion

Here we present a novel spinal fMRI study whereby we stimulate sensory dermatomes both above and below the level of traumatic spinal cord injury and employ a connectivity analysis to gauge how spinal circuits are altered along with sensory perception. We demonstrate that chronic incomplete SCI patients, when stimulated above the level of their injury, in a dermatome of normal sensation, show a heightened spinal fMRI response to thermal stimulation relative to controls (see figure 3, p = 0.025). This heightened response to thermal stimulation was not observed in SCI patients who fully recovered from their injury (ASIA E patients).

When we examined the relationship between the number of active voxels in a spinal cord region of interest corresponding to a dermatome of abnormal sensation, we demonstrated an inverse relationship between the degree of sensory impairment and the number of active voxels (see figure 4, R^2^ = 0.93, p<0.001). That is, thermal stimulation of dermatomes with a complete lack of sensory perception results in a higher number of active voxels in comparison to those with impaired sensation or normal sensation. Together, a significant increase in spinal fMRI response to thermal stimulation in dermatomes of normal sensation in incomplete SCI patients, a lack of this heightened response in persons who completely recover from their injury and a strong inverse correlation between the number of active voxels and the degree of sensory impairment support the notion of plasticity subsequent to traumatic spinal cord injury.

In order to capture the relationship between active voxels in the dorsal quadrant of the spinal cord corresponding to the dermatome stimulated (the prime cluster) and the remainder of active voxels in the spinal cord and brainstem we performed a connectivity analysis. Similar methods have been applied to brain fMRI data in order to understand the relationship between different brain regions that function together to produce complex attributes such as language[34] and how these networks may be disrupted in the presence of language difficulties such as stuttering.[35] To do this in the spinal cord, we employed a connectivity analysis whereby we looked for patterns between the prime cluster and either 1) the total number of connected voxels in the spinal cord and brainstem; 2) the total number of interspinal connections or 3) the total number of supraspinal connections. Our results indicate that the total number of connected voxels is not significantly different between controls and chronic SCI patients. However, there are a significantly increased number of interspinal connections in incomplete SCI patients relative to controls. Moreover, this increased degree of interspinal connectivity is maintained in patients who fully recover from their injury (figure 5). This change in pattern between controls and chronic SCI patients can be interpreted in terms of graph theory whereby the number of overall nodes in the spinal network is not significantly different (total number of connected voxels) but the connectivity of these nodes is different between controls and chronic SCI.

### Study Limitations

While unique in many respects, this study has certain limitations. We chose a chronic spinal cord injury population so as to avoid potential ongoing plasticity that often occurs within the first year of injury. However, we cannot be certain that our patient group is uniform with regard to circuit alternations after their respective injuries. Nonetheless, given that the majority of plastic changes subsequent to traumatic SCI are thought to occur in the first 12 months[5], an imaging study at one point in time across a total of 38 subjects does provide sufficient data to support the notion of an altered spinal cord response between groups. To build on these findings, it will be important to apply similar methods in a longitudinal fashion from the acute phase of injury through the chronic phase while conducting concordant clinical examinations to correlate to imaging parameters.

The inverse correlation between abnormal sensation in chronic, incomplete SCI patients with the number of active voxels in the corresponding region of the spinal cord (R^2^ = 0.93, p<0.001) is a rather robust finding in this study. Further characterizing this relationship will be important given that dermatomes of normal sensation above the level of injury in the same patient group show an overall higher average number of active voxels in the ROI analysis. Although this difference is not significant (p = 0.485) it is contrary to the inverse correlation observed in dermatomes of abnormal sensation whereby one might expect an overall lower number of active voxels in dermatomes of normal sensation. As can be gleaned from the electrophysiology literature, properties of spinal dorsal horn neurons can change dramatically in the setting of disrupted descending input either in experimental animal models[36] or in the setting of traumatic spinal cord injury. The data presented here suggest that with a greater degree of descending fiber disruption (more severe injury) caudal segments of the spinal cord respond in a more robust fashion to thermal stimulation (significantly higher number of active voxels). This finding agrees with electrophysiology data where it has been demonstrated that spinal cord transection in cats results in a higher firing rate of caudal dorsal horn neurons in response to heat stimuli.[36] The relationship between specific spinal cord segment response to stimulation and the degree of disruption of descending input through traumatic injury certainly warrants further investigation, as a graded stimulus-response-degree of injury relationship would further strengthen evidence for the specificity of the spinal fMRI signal.

### Conclusions

Spinal fMRI has overcome many technological challenges in recent years. This work represents the first of its kind to use innocuous heat stimulation across multiple sensory dermatomes in a single subject with spinal cord injury. We have demonstrated its feasibility and outlined specific metrics that can be used to quantify the spinal cord response. We have also highlighted particular limitations that with further study will allow for the advancement of this technology as a non-invasive means of determining the function of specific cell populations of the spinal cord (ROI analysis), how these cell populations work as a unit to transmit information to the brain (connectivity analysis) and how these circuits are altered after traumatic injury (spinal plasticity).
